# Energy metabolism and thermoregulation during sleep in young and old females

**DOI:** 10.1038/s41598-023-37407-3

**Published:** 2023-06-27

**Authors:** Jaehoon Seol, Chihiro Kokudo, Insung Park, Simeng Zhang, Katsuhiko Yajima, Tomohiro Okura, Kumpei Tokuyama

**Affiliations:** 1 International Institute for Integrative Sleep Medicine (WPI-IIIS), University of Tsukuba, 1-1-1 Tennodai, Tsukuba, Ibaraki Japan; 2 Research Center for Overwork-Related Disorders, National Institute of Occupational Safety and Health, Kawasaki, Japan; 3 R&D Center for Tailor-Made QOL, University of Tsukuba, Tsukuba, Japan; 4 Graduate School of Comprehensive Human Science, University of Tsukuba, Tsukuba, Japan; 5 Faculty of Pharmaceutical Sciences, Josai University, Saitama, Japan; 6 Institute of Health and Sports Sciences, University of Tsukuba, Tsukuba, Japan

**Keywords:** Ageing, Metabolism

## Abstract

Core body temperature (CBT) shows a diurnal rhythm, and the nocturnal decrease in CBT is blunted in older people. The physiological mechanisms responsible for the blunted nocturnal decrease in CBT in older people remain to be revealed. The aim of this study was to compare heat production and heat dissipation in young and old subjects during sleep, as assessed by indirect calorimetry and the distal–proximal temperature gradient (DPG) of skin temperature. A complete dataset of 9 young (23.3 ± 1.1 years) and 8 old (72.1 ± 2.5 years) females was analyzed. CBT and energy metabolism were monitored during sleep using an ingestible temperature sensor in a metabolic chamber maintained at 25 °C. Skin temperature was measured at proximal and distal parts of the body. CBT, distal skin temperature, and DPG in older subjects were higher than in young subjects. Protein oxidation was similar between the two groups, but fat oxidation was lower and carbohydrate oxidation was higher in old subjects compared to young subjects. On the other hand, energy expenditure was similar between the two age groups. Thus, the elevated CBT in older subjects was not attributed to deteriorated heat dissipation or enhanced heat production, suggesting an alternative explanation such as deteriorated evaporative heat loss in old subjects.

## Introduction

Core body temperature (CBT) shows a diurnal rhythm; CBT increases after awakening, peaks in the early evening followed by a gradual decrease, falls further after bedtime, and begins to increase prior to awakening^1–3^. Since body temperature is affected by food ingestion and physical activity as thermic effects of diet and exercise^4^, nocturnal sleep duration is the most reasonable period to assess CBT, free from these masking effects. The circadian rhythm amplitude of CBT is reduced in older people, mainly due to elevated CBT at night. The blunted decrease in nocturnal CBT was observed under real-life conditions^1–3^, during a non-entrained (free-running) condition^1^, and also in a constant routine protocol^3^. The physiological mechanisms responsible for the blunted nocturnal decrease of CBT in older people remain to be clarified.

Energy expenditure and body temperature affect each other; energy expenditure as heat production, and body temperature affect energy metabolism through the Q_10_ effect^5^. The Q_10_ for biological reactions is 2.0–3.0, and it is expected that a 1 °C rise in body temperature increases the rate of chemical reactions, including energy metabolism, by 7–12%^6^. In a classical study, Du Bois showed that a 1 °C increase in body temperature was associated with approximately a 13% increase in metabolic rate in patients with a fever episode^7^. Analysis of daily energy expenditure through the human life course, using the doubly labeled water method in free-living conditions, shows that daily energy expenditure is stable from ages 20 to 60, and it begins to decline after 60 years of age^8^. However, it is not known whether sleep metabolic rate is also reduced in aged subjects.

Indirect calorimetry estimates the oxidation of macronutrients: protein, fat, and carbohydrate. Protein catabolism is estimated from urinary nitrogen excretion, and fuel selection between fat and carbohydrate is reflected as non-protein respiratory quotient (RQ), i.e., the ratio of CO_2_ production to O_2_ consumption, which does not arise from protein oxidation. A higher RQ implies dominant carbohydrate oxidation, and a lower RQ reflects dominant fat oxidation^9^. RQ increases in response to a meal during the daytime and decreases during sleep, which defines the range of RQ over 24 h, i.e., one of the definitions of metabolic flexibility^10^. In young subjects, oxidized substrate shifts from carbohydrate to fat, reflected as a decrease in RQ during the first half of sleep, but RQ begins to increase prior to awakening, suggesting that energy metabolism during sleep is not a simple extension of energy metabolism during fasting^10,11^. When energy metabolism during sleep was compared between two groups with a 10-year difference, the sleep RQ of the older subjects (32.5 ± 2.0 years) was significantly higher than that of the younger subjects (22.5 ± 0.2 years). Older subjects showed slightly lower energy expenditure compared to subjects 10 years younger, although the difference was not statistically significant^10^. Taken together, it is expected that age-related differences in energy expenditure and substrate oxidation during sleep will become more pronounced when comparing two groups further apart in age.

There is a paucity of literature on sleeping energy metabolism and body temperature in older female subjects. The aim of the present study was to identify age-related changes in energy metabolism and thermoregulation during sleep, comparing two female groups 50 years apart.

## Results

### Characteristics of participants

Physical characteristics are shown in Table 1. Compared with young subjects, older subjects were shorter and had a higher BMI, but body weight, body fat %, and lean body mass were not significantly different between the two age groups. According to "the National Health and Nutrition Survey in Japan, 2019," the average height, weight, and BMI for ages 20–29 are 157.5 ± 5.5 cm, 52.0 ± 8.1 kg, 21.0 ± 2.9 kg/m^2^, and for ages 70–74 are 151.4 ± 5.4 cm, 52.9 ± 8.2 kg, 23.1 ± 3.5 kg/m^2^, respectively^12^. The participants were considered to represent their age groups well. The habitual bedtime of older subjects was about 2 h earlier than that of young subjects.

**Table 1:** Characteristics of the participants.

	Young (n = 9)	Old (n = 8)	*p* value
Age (years)	23.3 ± 1.1	72.1 ± 2.5	< 0.001
Height (cm)	161.8 ± 5.5	152.3 ± 5.4	0.003
Weight (kg)	53.9 ± 9.3	53.8 ± 4.8	0.978
BMI (kg/m^2^)	20.5 ± 2.6	23.3 ± 2.4	0.038
Body fat (%)	27.6 ± 5.7	31.2 ± 3.1	0.137
LBM (kg)	38.7 ± 4.5	36.9 ± 2.0	0.307
PSQI-J (points)	5.6 ± 2.3	5.4 ± 1.8	0.778
Habitual bedtime (h:min)	0:03 ± 1:04	22:06 ± 0:45	< 0.001

Values are shown as means ± SD. *BMI* body mass index, *LBM* lean body mass. *PSQI-J* The Japanese version of the Pittsburgh Sleep Quality Index.

### Body temperatures

**Figure 1:**
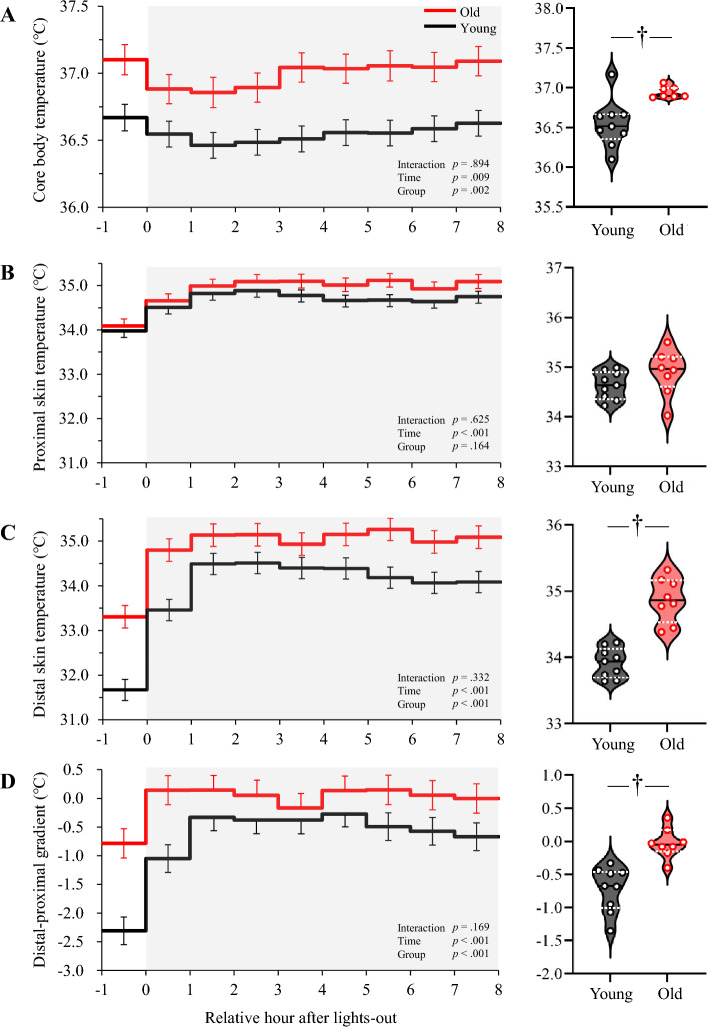
Time course of body temperatures and average temperatures during sleep. The time course shows the core body temperature from 1 h before lights-out to light-on (left panel) and the average value during sleep (right panel) (**A**), proximal skin temperature (**B**), distal skin temperature (**C**), and distal–proximal gradient (**D**). The time course is shown as means ± SE. The black solid line and white dashed lines in the violin plot represent the median and the 25th and 75th percentiles, respectively. † represents a significant difference between young and old (*p* < 0.05).

CBT of the old subjects was higher than that of young subjects during the entire period (Fig. 1A). There was no significant difference in the batiphase of CBT (2:43 ± 0:50 for young, 1:00 ± 0:56 for old subjects, *p* = 0.190). Although there was no group difference in proximal skin temperature (Fig. 1B), distal skin temperature (Fig. 1C) and DPG (Fig. 1D) were higher in old subjects compared to young subjects.

### Energy metabolism

**Figure 2:**
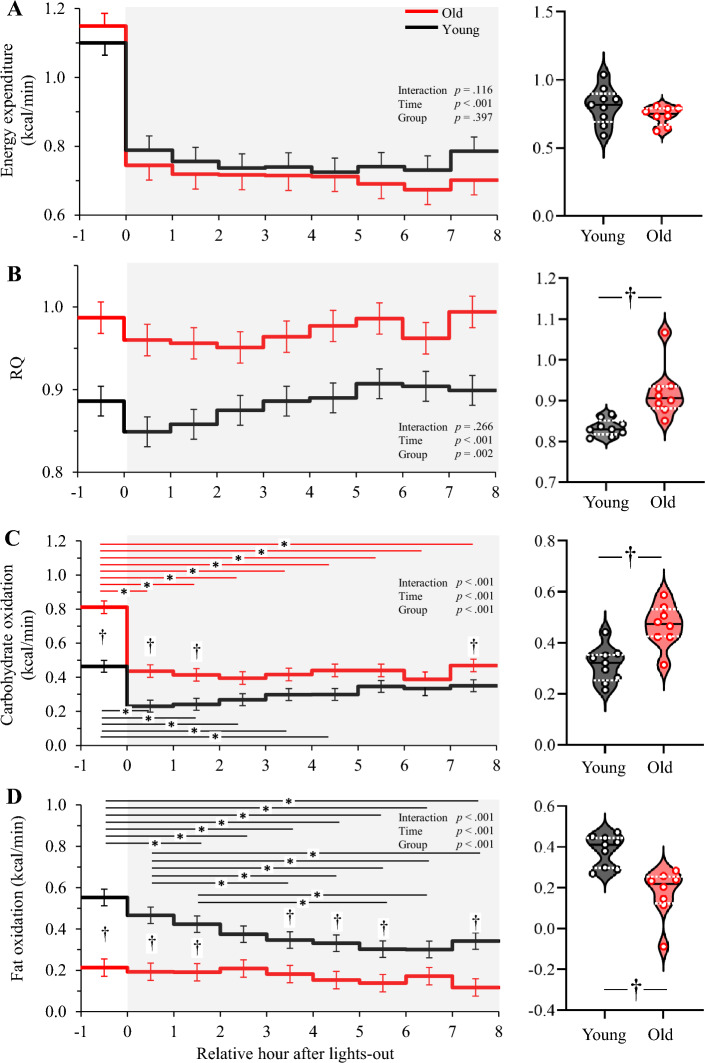
Time course of energy metabolism and average metabolism during sleep. The time course shows the energy expenditure from 1 h before lights-out to light-on (left panel) and the average value during sleep (right panel) (**A**), RQ (**B**), carbohydrate oxidation (**C**), and fat oxidation (**D**). The time course is shown as means ± SE. The black solid line and white dashed lines represent the median and the 25th and 75th percentiles, respectively. * represents a difference between time points (*p* < 0.05). † represents a difference between young and old (*p* < 0.05).

There was no significant difference in energy expenditure adjusted for lean body mass (LBM) between the two age groups (Fig. 2A). Old subjects had a higher RQ than young subjects (Fig. 2B). Carbohydrate oxidation of old subjects was higher than that of young subjects. Compared to 1 h before bedtime, it was lower during sleep, especially in the first half of sleep, in both age groups (Fig. 2C). Fat oxidation was lower in older subjects than in young subjects. Fat oxidation in young subjects decreased during sleep, whereas fat oxidation in old subjects remained at the same level as 1 h before bedtime throughout the entire period (Fig. 2D).

### Relationship between fat oxidation and body composition

Fat oxidation was positively correlated with LBM in young (*r* = 0.87, *p* = 0.002), old (*r* = 0.60, *p* = 0.114), and both age groups combined (*r* = 0.60, *p* = 0.011). Fat oxidation was positively correlated with body fat within the old group (*r* = 0.74, *p* = 0.036), but the correlation was not significant in the young group (*r* = 0.50, *p* = 0.174) and in all subjects (*r* = 0.22, *p* = 0.396).

### Protein catabolism

Protein oxidation, assessed from urinary excretion of nitrogen, did not differ between the two age groups (54.2 ± 5.5 kcal/8 h for young, 45.5 ± 5.5 kcal/8 h for old, *p* = 0.282).

## Discussion

In the present study, CBT during sleep in old subjects was higher than that of young subjects, which is consistent with previous studies^1–3^. The hourly average of CBT was lowest in the second hour after bedtime in both young and old subjects. Given the 2-h earlier lights-off for old subjects, the diurnal rhythm of CBT in old subjects seems to be advanced compared to that of young subjects. However, the difference in batiphase of CBT between young subjects (2:43) and old subjects (1:00) was not statistically significant, probably due to the low statistical power of the experiment. In the present study, measurements for young females were performed during the follicular phase. It is noteworthy that the batiphase of CBT is not affected by the menstrual cycle or the use of oral contraceptives^13–15^.

There are two previous studies that oppose our findings. First, one study reported lower CBT during sleep in postmenopausal women (56.2 ± 1.6 years) than that of premenopausal women during the follicular phase (32.0 ± 1.7 years)^16^. A plausible explanation for this discrepancy is the difference in environmental temperature during the measurement, which was 25 °C in the present study, whereas it was 21 °C in the previous study^16^. It is known that older subjects have difficulty in maintaining CBT at a lower environmental temperature. When the room temperature was gradually lowered from 26.5 to 20 °C, young subjects kept their CBT unchanged, while older subjects could not keep their CBT and it gradually decreased^17^. The deteriorated adaptation ability and reduced capacity for brown fat thermogenesis^18^ would make the CBT of older subjects easily affected by lower environmental temperature. The second study is about the timing of the batiphase of CBT. It was observed in the first half of the sleep period in the present study and other reports^13–15^, while a study on long-term CBT measurements in female subjects under real-life conditions reported interindividual variation, and it was observed in the second half of sleep in some cases^19^. Further studies are required to identify the possible cause responsible for the individual variation in the time course of CBT during sleep.

The elevated CBT during sleep in older subjects was not associated with an increase in energy expenditure, i.e., heat production. An increased energy expenditure, as the Q_10_ effect predicted, was not observed in old subjects. Another possibility explaining the elevated CBT in old subjects is impaired heat dissipation. However, DPG of old subjects was higher than that of young subjects, and the higher CBT of older subjects during sleep cannot be attributed to the downregulation of heat dissipation from peripheral parts of the body. The elevated DPG observed in old subjects was mainly due to higher distal skin temperature compared to young subjects. The higher distal skin temperature in old subjects in the present study may be related to age-related changes in the physiological function of the body and/or behavior. Although all participants used the same blanket and bed in the chamber maintained at 25 °C, it was not known if their extremities had been inside or outside the blanket during sleep. Heat loss from the distal skin is assessed by DPG, which is based on vasodilation and blood flow, and its mechanisms are considered as heat dissipation by convection, conduction, and radiation. If those mechanisms are working but CBT is still elevated, then one of the possible remaining factors is a problem of evaporative heat loss such as perspiration. Attenuated evaporative heat loss of older subjects in a hot environment and during exercise results in higher body temperature^20,21^, but age-related changes in evaporative heat loss during sleep remain to be identified.

It is well known that sleep quality deteriorates with aging^1,2,22,23^: lower sleep efficiency because of increased arousal and shorter slow-wave sleep. Poor sleep quality in older subjects may be related to their higher CBT during sleep observed in the present study. Besides aging, the association of poor sleep quality and nocturnally elevated CBT has been observed in patients with insomnia^24^ and affective disorder^25^. The elevated nocturnal CBT in older subjects^3^ and in poorer sleepers^26^ is also observed when sleep is prohibited during a constant routine protocol: subjects are not allowed to sleep, kept in constant light and temperature, and maintain a constant semi-recumbent posture for the duration. These observations suggest that elevated nocturnal CBT may underlie poor sleep quality. This causal relationship is consistent with the fact that hypothalamic areas involved in regulating body temperature and sleep are anatomically overlapping in the preoptic area (POA)^27^.

The time course of RQ was significantly different between the two age groups 50 years apart in the present study, which reinforced our previous results comparing two groups with 10 years apart^10^. The significantly different BMI between the two groups might have a potential impact on RQ since nocturnal decrease of RQ is smaller in subjects with higher BMI^28^. According to the criteria of judgement of the Japan Society for the Study of Obesity, a BMI score less than 18.5 kg/m^2^ is categorized as underweight and equal to or more than 25.0 kg/m^2^ as overweight, and one young participant and two old participants fall in those categories, respectively. However, there was no correlation between RQ and BMI (*r* = 0.10, *p* = 0.702) and between RQ and body fat percentage (*r* = 0.13, *p* = 0.611). The decline in fat oxidation in older subjects is consistent with most studies reporting an age-dependent decrease in fat oxidation under various conditions: in a postabsorptive state measured using hood-type calorimetry^29–31^ and average value over 24 h using a metabolic chamber^32,33^. The blunted nocturnal decline in RQ was observed in subjects with metabolic inflexibility, defined as smaller range of diurnal changes in RQ in young subjects without obesity^10^ and with obesity^34^. Although the present study did not follow diurnal changes in RQ over 24 h, the blunted nocturnal decline in RQ in older subjects suggests that metabolic flexibility deteriorates with aging.

There are several limitations in this study. First, heat production in the present study was assessed by indirect calorimetry, which does not account for anaerobic respiration of gut microbiota. It has been suggested that the microbiota produces heat and affects the temperature of the host^35^. Second, subjects consumed dinner 5 h before bedtime, but the composition of the meal was at their own choice and not analyzed, which might affect the energy expenditure and substrate oxidation. Third, more studies on both males and females in their 30 s, 40 s, 50 s, 60 s, and older are required to generalize age-related changes in energy metabolism and thermoregulation.

## Methods

### Determination of sample size

The sample size was determined using G*power version 3.1, based on an effect size of 0.25, α level of < 0.05, and 80% power. The result indicated that the total sample size was 16. Assuming missing values due to operational mistakes and/or malfunctions of measurement apparatus, an additional number of subjects were recruited through advertisements: 9 young and 9 old females.

### Participants

All subjects were not taking sleeping pills, non-smokers, non-shift workers, and had no drinking habit more than three times a week, and no exercise habit more than two times a week for the past 6 months. They had not been diagnosed with insomnia/sleep apnea syndrome and had not engaged in trans meridian travel within 1 month prior to the experiment. In addition, young participants were required to have a normal menstrual cycle and had no history of taking contraceptive pills. The experiment for young participants was carried out in the early follicular phase of the menstrual cycle. The purpose and risks were explained, and the informed written consent was obtained from all the subjects. The study was approved by the ethics committee of the University of Tsukuba (Ref No., Tai 29-29 and Ref No., Tai 30-134) and performed in accordance with the principles of the Declaration of Helsinki.

### Protocol

The subjective sleep quality was assessed using the Japanese version of the Pittsburgh Sleep Quality Index (PSQI-J)^36,37^, which was administered before the indirect calorimetry. Habitual bedtime was asked in the pre-study interview, and the participants were encouraged to keep their regular wake-sleep schedule and eating habits for 1 week before the experiment day. Their wake-sleep schedule was confirmed by actigraphy recordings (Supplemental Fig. 1). The sleeping period was set as 8 h from the habitual bedtime, and the participants were required to stay in bed until the lights in the chamber turned on. The experiment was preceded by an adaptation night in the metabolic chamber, during which the sensors of the recording system were attached to the participants. They were also instructed to refrain from taking caffeine and alcohol at least 2 days before the experiment day.

On the experimental day, subjects took their usual dinner and arrived at the laboratory 5 h and 3 h prior to their habitual bedtime, respectively. All sensors were attached to the subject, and they were instructed to urinate before entering the metabolic chamber 1 h prior to their habitual bedtime. Subjects were instructed to remain sedentary in the chamber, and urine was collected during the indirect calorimetry. Energy metabolism was measured from 1 h prior to the habitual bedtime to wake-up time the next morning.

### Energy metabolism

Energy expenditure, RQ, and oxidation of macronutrients were measured by an indirect calorimetry using a metabolic chamber. The airtight chamber, measuring 2.00 × 3.45 × 2.10 m with an internal volume of 14.49 m^3^, was furnished with a mattress, desk, chair, and toilet. The temperature and relative humidity in the chamber were controlled and maintained at 25.0 ± 0.5 °C and 55.0 ± 3.0%, respectively^10^.

Concentrations of oxygen (O_2_) and carbon dioxide (CO_2_) in the chamber were measured with online process mass spectrometry (VG Prima δB, Thermo Electron, Winsford, UK). O_2_ consumption (${\dot{\text{V}}}$O_2_) and CO_2_ production (${\dot{\text{V}}}$CO_2_) rates were calculated using an algorithm for improved transient response^38^. Energy expenditure and macronutrient oxidation were calculated based on ${\dot{\text{V}}}$O_2_, ${\dot{\text{V}}}$CO_2_, and urinary nitrogen excretion, and the RQ was determined as the ratio of CO_2_ production to O_2_ consumption, which did not arise from protein oxidation, known as non-protein RQ^9^. The body composition was measured using the bioimpedance method (BC-118E, TANITA Co., Tokyo, Japan).

### Thermometry

CBT was measured with an ingestible sensor and wireless data recorder (CorTemp, HQ, Inc., FL, USA). The batiphase of CBT was defined as the time when the lowest 1-h average of CBT was observed. Skin temperatures were continuously monitored at eight sites: midforehead, 1 cm above the navel (stomach), right infraclavicular area, midthigh on the right musculus rectus femoris, the center of the middle back of the left and right hand (later averaged), and the middle of the left and right foot instep (later averaged). Thermistor probes (ITP082-24, Nikkiso-Thermo Co., Tokyo, Japan) connected to a data logger (N543, Nikkiso-Thermo Co.) were fixed to the skin with thin air-permeable adhesive surgical tape. The average proximal temperature was calculated using the equation: 0.093 forehead + 0.347 thigh + 0.266 infraclavicular area + 0.294 stomach. The average distal skin temperature was calculated from the mean of both hands and feet. To assess heat dissipation, DPG of skin temperature was calculated as the difference between the distal and proximal skin temperature^39^.

### Statistical analysis

Data from one old female were not incorporated due to a technical problem. A complete dataset from 9 young and 8 old participants was analyzed. Outcomes of interest are presented as means ± SE, except for characteristics of participants, which are shown as means ± SD. Characteristics of participants, batiphase of CBT, and protein catabolism of the two age groups were analyzed by an unpaired *t* test. The time course of energy expenditure was adjusted for LBM. A mixed model for repeated measures (GLMM) was selected since the hourly average in energy metabolism, CBT, and skin temperature were not normally distributed (all p < 0.05). Holm’s post-hoc tests were used to correct for multiple comparisons. Pearson's correlation coefficient was used for correlation analysis. All statistical analyses were conducted using IBM SPSS statistical software Version 28.0 (IBM Japan, Ltd., Tokyo, Japan). Statistical significance was set at 0.05 (two-tailed).

## Supplementary Information

Supplementary Figure 1.

## Data Availability

The datasets used and/or analyzed during the current study are available from the corresponding author upon reasonable request.
