# Supplementary material for: Energy metabolism and thermoregulation during sleep in young and old females
Source: Sci Rep. 2023 Jun 27;13:10416. doi: 10.1038/s41598-023-37407-3 (PMC10299995; doi:10.1038/s41598-023-37407-3)
Supplement: Supplementary file 1 — Supplementary Figure 1. [file 41598_2023_37407_MOESM1_ESM.pdf]

**Energy metabolism and thermoregulation during sleep in young and old females**

Jaehoon Seol[1,2,3†], Chihiro Kokudo[1,4†], Insung Park[1], Simeng Zhang[1], Katsuhiko Yajima[5], Tomohiro Okura[1,2,6], Kumpei Tokuyama[1*]

Supplementary Information

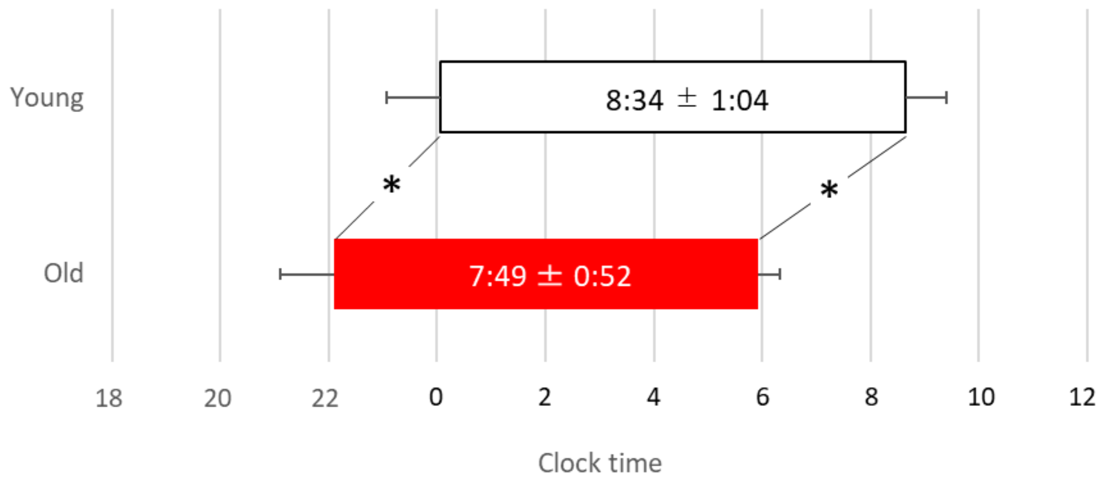


Supplemental Figure 1. Habitual bedtime and wake-up time prior to the study

Habitual bedtime and wake-up time were analyzed based on actigraphy recordings lasting for about 1 week (5-7 days) including weekend. Total bedtime was calculated as difference between bedtime and wake time. * significant difference between young and old ($p < 0.05$).
